# Prevalence and factors associated with non-alcoholic fatty liver disease among women with polycystic ovary syndrome

**DOI:** 10.61622/rbgo/2024rbgo81

**Published:** 2024-12-04

**Authors:** Maria Elisa Franciscatto, Juliana Bosso Taniguchi, Raquel Wohlenberg, Isadora Luísa Riedi, Karen Oppermann

**Affiliations:** 1 Universidade de Passo Fundo Faculty of Medicine Passo Fundo RS Brazil Faculty of Medicine, Universidade de Passo Fundo, Passo Fundo, RS, Brazil.; 2 Hospital São Vicente de Paulo Gynecology and Obstetrics Passo Fundo RS Brazil Gynecology and Obstetrics, Hospital São Vicente de Paulo, Passo Fundo, RS, Brazil.

**Keywords:** Non-alcoholic fatty liver disease, Polycystic ovary syndrome, Metabolic syndrome, Alcohol drinking, Obesity, Waist circumference, Hyperandrogenism

## Abstract

**Objective:**

To verify the prevalence and factors associated with Non-Alcoholic Fatty Liver Disease (NAFLD) among women with Polycystic Ovary Syndrome (PCOS).

**Methods:**

A cross-sectional study was conducted with 53 patients with PCOS. The diagnosis of PCOS followed the Rotterdam criteria. The diagnosis of NAFLD was made through US showing hepatic steatosis, excluding significant alcohol consumption and chronic liver disease. The following variables were compared between the groups of women with and without NAFLD: age, race, anthropometric data, blood pressure levels, liver enzymes, glycemic and lipid profiles, total testosterone, presence of hirsutism, and metabolic syndrome (MS). Variables were compared between the groups using T-test, Mann-Whitney, and Chi-square tests.

**Results:**

Among 53 patients with PCOS, 50.9% had NAFLD. The NAFLD group had higher weight (p=0.003), BMI (p=0.001), waist circumference (p≤0.001), fasting glucose (p=0.021), HbA1C% (p=0.028), triglycerides (p=0.023), AST (p=0.004), ALT (p=0.001), higher prevalence of MS (p=0.004), and lower levels of HDL cholesterol (p=0.043). The other variables did not differ between the groups. Both groups were predominantly of caucasian race, and there was no significant difference in age.

**Conclusion:**

The prevalence of NAFLD among patients with PCOS was 50.9%. Metabolic and hepatic enzyme abnormalities were more prevalent in this group compared to the group without the disease. Obesity tripled the prevalence of NAFLD.

## Introduction

Polycystic ovary syndrome (PCOS) is one of the most common reproductive endocrine disorders in women.^(1)^ Its prevalence ranges from 9 to 18%, varying according to the diagnostic criteria employed and the studied population.^(2)^ The Rotterdam criteria are the most used and described in the literature for the diagnosis of PCOS.^(3)^ In 2023, there was an update in which the presence of irregular cycles together with clinical hyperandrogenism, excluding other causes, establishes the diagnosis. In the absence of clinical hyperandrogenism, biochemical evaluation is recommended to define the diagnosis and exclude other causes. In the presence of only irregular cycles or hyperandrogenism, ultrasound or measurement of anti-Mullerian hormone levels in adult women is suggested. Hyperandrogenism is responsible for the characteristic clinical manifestations: hirsutism, acne, oily skin, alopecia, and virilization.^(3)^ Furthermore, obesity and insulin resistance are strongly present in this syndrome, and both are considered to play essential roles in the pathophysiology of PCOS. Hyperinsulinemia stimulates androgen production by the ovaries and is reinforced by bidirectional links between insulin resistance and hyperandrogenism.^(4)^ Women with this disorder have an established increased risk of developing type 2 diabetes and a still debated increased risk of cardiovascular disease.^(5)^ The global increase in the prevalence of obesity and insulin resistance has brought metabolic and health-related repercussions, including NAFLD, in various populations.^(6–8)^

NAFLD is the most common chronic liver disease worldwide, characterized by fat accumulation in the liver tissue without inflammation.^(6)^ Although less prevalent in this presentation of liver disease, NAFLD can progress to steatohepatitis (accumulation of fat in liver tissue with inflammation and hepatocellular damage), liver cirrhosis, and possibly hepatocellular carcinoma. PCOS and NAFLD share similar metabolic changes, such as obesity and insulin resistance, as well as cardiovascular diseases and type 2 diabetes mellitus.^(7)^ Currently, studies have been linking elevated serum levels of androgens as independent predictors of NAFLD in women with PCOS,^(9–31)^ but this is not shared by other authors.^(27,32)^ The intrinsic association of obesity and insulin resistance with NAFLD may coexist with hyperandrogenism, a frequent characteristic in patients with PCOS, making it difficult to understand the exact role of each of these factors in NAFLD among patients with PCOS.

The present study aims to verify the prevalence and factors associated with NAFLD in patients with PCOS seen at an Endocrine Gynecology outpatient clinic in the interior of the state of Rio Grande do Sul.

## Methods

This is a cross-sectional study, conducted with 53 patients seen at the Polycystic Ovary Syndrome Clinic of Hospital São Vicente de Paulo (HSVP) in Passo Fundo, Rio Grande do Sul. The personal information, clinical, laboratory, and ultrasound data of the patients were collected between January 2015 and January 2022.

Patients were referred to the PCOS outpatient clinic due to a variety of complaints, including menstrual irregularity, hirsutism, alopecia, acne, and/or a desire to become pregnant. The diagnosis of PCOS was made according to the Rotterdam criteria (2004 update), which included at least two of the following criteria: anovulation, hyperandrogenism (clinical and/or laboratory), and polycystic ovarian morphology on ultrasound (presence of at least one of the following findings: 12 or more follicles measuring between 2-9 mm in diameter or ovarian volume greater than 10 cm³ in at least one of the ovaries), excluding other causes of hyperandrogenism such as hyperprolactinemia, non-classical congenital adrenal hyperplasia, thyroid dysfunction, Cushing's syndrome, and androgen-secreting neoplasms.^(3)^ For all patients diagnosed with PCOS, an abdominal ultrasound was requested.

The diagnosis of hepatic steatosis was made by abdominal ultrasound in the HSVP Radiology department, through the increased echogenicity of the liver parenchyma compared to the renal cortex and the greater attenuation of the acoustic beam.^(12)^ For the characterization of NAFLD, significant alcohol consumption (> 70g/week for women) was considered an exclusion factor.^(13)^

The following variables were verified in the group of PCOS women: age in years, race, weight, height, Body Mass Index (BMI), Waist Circumference (WC), Systolic Blood Pressure (SBP), Diastolic Blood Pressure (DBP), degree of hirsutism, and presence of Metabolic Syndrome (MS).

Body weight was measured on a digital scale with the patient without shoes and coats, standing in the center of the scale, weight equally distributed on both feet, recorded in kg. The height was measured with the patient barefoot in an anatomical position with calves, buttocks, shoulders, and head touching the vertical surface of the measuring device, with the support positioned over the head, pressing only the hair. The measurement was recorded in meters. BMI was calculated using the formula $\text{BMI}=\text{weight}(\text{kg})/\text{height}(\text{m}{)}^{2}$ and classified into three categories: normal weight (18.5-24.9 kg/m²), overweight (25-29.9 kg/m²), and obesity (≥30 kg/m²). WC, in cm, was measured at the midpoint between the lower rib margin and the iliac crest.^(14)^ Blood pressure measurements for SBP and DBP, in mmHg, were taken with the patient seated and at rest, using an appropriately sized cuff positioned 2 to 3 centimeters above the cubital fossa.^(15)^ The degree of hirsutism was assessed using the Ferriman-Gallwey score, considering a score of 8 or higher as indicative of hirsutism. MS was defined by the presence of at least three of the following abnormalities: waist circumference ≥ 88 cm, fasting glucose ≥ 100 mg/dL, triglycerides ≥ 150 mg/dL, HDL cholesterol < 50 mg/dL, and blood pressure ≥ 130/85 mmHg or being treated for systemic arterial hypertension.^(11)^

Laboratory variables were collected from blood samples from a peripheral vein in the upper limb after a 12-hour fast in the morning for hormonal and biochemical measurements. The following tests were measured in each blood sample: AST/TGO (aspartate aminotransferase) and ALT/TGP (alanine aminotransferase) in U/L, glycated hemoglobin (HbA1c) in percentage, fasting blood glucose (FG) in mg/dL, total cholesterol, HDL cholesterol, triglycerides (TG) in mg/dL, and total testosterone (TT) in ng/dL or nmol/L.

The following variables were analyzed dichotomously: hirsutism (Ferriman score ≥8), laboratory hyperandrogenism (total testosterone > 60 ng/dL or >2.1 nmol/L), race (caucasian), metabolic syndrome (MS: yes), waist circumference ≥ 88 cm, and BMI ≥ 30 kg/m².

In statistical analysis, variables were described as mean, median, or percentage. For numerical variables, normality was tested using the Kolmogorov-Smirnov test. For variables with normal distribution, the Student's t-test was applied, and for non-normal variables, the Mann-Whitney U test was applied. In comparison tests between groups with or without NAFLD, the chi-square test and Fisher's exact test were used for qualitative variables. The prevalence ratio and Poisson regression of variables associated with NAFLD were calculated. A significance level of p < 0.05 was considered.

## Results

The prevalence of NAFLD among the 53 PCOS patients studied was 50.9%. There was a predominance of the caucasian race. The average age of the general group was 25 ± 6.9 years, with a tendency for the group with NAFLD to be older than the group without NAFLD (p = 0.07), as shown in table 1. Of the 53 participants, 64% classified themselves as obese (BMI ≥ 30 kg/m²), 24.5% were overweight (BMI between 25 and 29.9 kg/m²), and 11% had a normal BMI (BMI < 25 kg/m²).

**Table 1:** Anthropometric, clinical, and laboratory characteristics of PCOS patients with and without NAFLD

Variable	PCOS with NAFLD n = 27	PCOS without NAFLD n = 26	p-value
Age, years	29.0 ± 6.6	25.6 ± 6.7	0.070
Caucasian race, %	70.8	83.3	0.494
Weight, kg	92.7 ± 20.6	79.8 ±14.5	0.003
BMI, kg/m^2^	36.9 ± 6.5	30.7 ± 6.5	0.001
WC, cm	108.7 ± 13.7	92.5 ± 14.7	0.001
SBP, mmHg	128 ± 14.2	120 ± 17.7	0.130
DBP, mmHg	83 ± 9.3	80 ± 13.8	0.187
HbA1c, %	6.2 ± 2	5.2 ± 0.5	0.028
MS, %	80.8	38.5	0.004
AST/GOT, U/L (mean ± DP)	26.8 ± 16.8	17.9 ± 3.9	0.004
ALT/GPT, U/L (mean ± DP)	30.7 ± 19.5	17 ± 7.4	0.001

BMI: body mass index; SBP: systolic blood pressure; DBP: diastolic blood pressure; MS: metabolic syndrome; WC: waist circumference

The group of patients with NAFLD had greater body weight (p = 0.003), greater waist circumference (p < 0.001), higher fasting blood glucose (p = 0.021), higher HbA1c levels (p = 0.028), lower HDL cholesterol levels (p = 0.043), higher triglyceride levels (p = 0.023), higher levels of AST/ALT (p = 0.004) and ALT (p = 0.001). As expected, the NAFLD group had a significantly higher prevalence of metabolic syndrome, 80.8% (p = 0.004), compared to the group without NAFLD, 38.5%.

**Figure 1:**
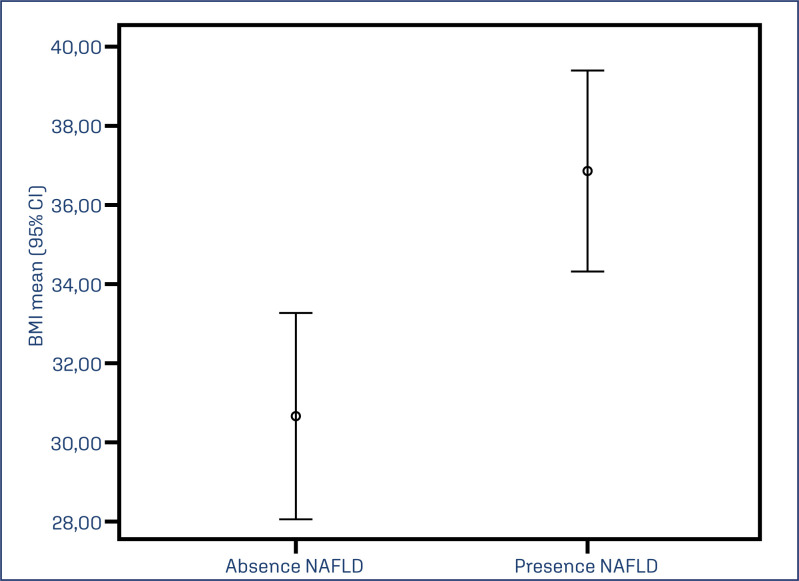
Relationship between BMI (Kg/m²) and NAFLD among patients with Polycystic Ovary Syndrome (p=0.024) with prevalence ratio and 95% confidence interval (95% CI)

Other parameters were not statistically different between the groups with and without NAFLD (Table 1). In figure 1, a graphical relationship is presented between BMI and the presence or absence of NAFLD in patients with PCOS. A higher mean BMI was observed in the group of patients with NAFLD (36.9 kg/m²) compared to the group without NAFLD (30.7 kg/m²), p=0.001. Table 2 describes the association of BMI and WC for NAFLD. For this group of patients, obesity (BMI ≥ 30 kg/m²) significantly increased the risk of NAFLD: 3.21 (95% CI: 1.31 – 7.91), even after controlling for hyperandrogenism. The association of waist circumference also increased the risk of NAFLD; however, it was not significant for this group of patients.

**Table 2:** Prevalence ratio and 95% confidence interval (95%CI) of BMI and WC for NAFLD

Univariate model - BMI	3.21 (95%CI: 1.31 – 7.91)
Univariate model - WS	3.27 (95%CI: 0.91– 11.75)
Model - BMI controlled for laboratory hyperandrogenism	3.16 (95%CI: 1.28 – 7.81)

* laboratory hyperandrogenism: total testosterone >60ng/dl or >2.1nmol/L

## Discussion

The prevalence of NAFLD in patients with PCOS is quite variable in the literature, most likely due to different study designs, PCOS diagnostic criteria, and diverse populations. In the present study, the prevalence of NAFLD in women with PCOS was 50.9%, while reports in the literature vary between 38% and 69%.^(20)^

The risk of having NAFLD among women with PCOS was verified in a large study of 50 785 354 women. The risk of NAFLD was significantly higher in this group of women (OR 4.30, 95% CI 4.11 to 4.50, p < 0.001).^(17)^

The factors associated with this pathology exhibit variability across studies; however, the majority of literature delineates a correlation between NAFLD and PCOS, involving Insulin Resistance (IR), central obesity, and hyperandrogenism.^(30–32)^ Regardless, there is a consistent elevation in NAFLD risk among women with PCOS compared to those without.^(20)^ Additionally, it is proposed that NAFLD should not be regarded merely as an isolated ultrasound finding but rather as an integral hepatic component of Metabolic Syndrome (MS) due to their closely intertwined nature.^(29)^ These findings substantiate the outcomes of our study, wherein the cohort of women with NAFLD exhibited a markedly heightened prevalence of MS.

Regarding the pathophysiology of NAFLD and PCOS, it is widely acknowledged that the prevailing connection between these two pathological conditions is Insulin Resistance (IR). Hyperinsulinemia instigates a decline in mitochondrial oxidation of fatty acids, fostering inflammation, necrosis, fibrosis, and consequent NAFLD progression.^(22)^ It is estimated that IR manifests in approximately 80% of NAFLD patients, with insulin levels serving as an independent determinant of NAFLD presence.^(21)^ The pivotal role of IR in PCOS is also firmly established,^(23)^ with a prevalence reaching up to 80% in PCOS patients with concurrent NAFLD.^(26)^ Pioneering research has correlated IR with PCOS among overweight and obese individuals,^(24)^ although discerning whether IR ensues as a consequence of excessive weight or PCOS remains challenging. Nonetheless, contemporary evidence has convincingly elucidated the IR mechanism even in lean women with PCOS.^(25)^

The current study lacked an adequate sample size to assess specific Insulin Resistance (IR) markers, such as the Homeostatic Model Assessment of Insulin Resistance (HOMA-IR) index and the Oral Glucose Tolerance Test (OGTT). Incorporating such markers would strengthen the correlation between IR and NAFLD in PCOS. Nevertheless, individuals diagnosed with NAFLD in our study exhibited significantly larger waist circumferences, a clinical indicator reflecting intra-abdominal fat accumulation, thereby serving as an indirect measure of IR.

Some studies have indicated that hyperandrogenism, which is also associated with insulin resistance and obesity,^(28)^ contributes to the onset of NAFLD in women with PCOS. However, it remains unclear whether androgens directly impact the development of NAFLD in PCOS or exert an indirect influence through insulin resistance. Dysregulation of insulin signaling in the ovaries of women with PCOS results in heightened androgen secretion, while insulin resistance concurrently diminishes sex hormone synthesis and elevates free androgen levels.^(32)^ Androgens, in turn, downregulate LDL receptor gene expression, thereby heightening the risk of hepatic steatosis.^(30)^

The association between isolated hyperandrogenism and NAFLD remains a subject of contention in the literature. Some studies indicate this association; for instance, a case-control study found that hyperandrogenism was linked to NAFLD even after adjusting for age, BMI, lipid profile, insulin resistance (IR), or glycemic status, suggesting it serves as an independent risk factor for NAFLD in women with PCOS and those who are not obese.^(31)^ Additionally, a cross-sectional study involving 400 Chinese women demonstrated that elevated levels of free androgen are correlated with NAFLD in women with PCOS, irrespective of obesity and/or insulin resistance presence.^(30)^ This association was corroborated in a meta-analysis comprising 17 studies;^(19)^ however, few of these studies exhibited substantial heterogeneity.

The association between NAFLD and obesity among PCOS patients is firmly established. Studies have reported on this association, demonstrating its independence from androgen levels and reliance solely on obesity and central fat deposition, attributes that are prevalent among women with PCOS.^(27,32)^

The present study revealed a pronounced prevalence of obesity, with even higher BMI levels detected among patients with NAFLD, thus underscoring this robust association. In addition to obesity, central fat deposition emerged as the primary independent factor associated with NAFLD in this cohort of PCOS patients. Notably, no association between hyperandrogenism and NAFLD was observed in this sample.

As limitations of this study, it must be noted that the sample size was insufficient to elucidate the relationship between hyperandrogenism and NAFLD, necessitating an expansion of the sample for a more comprehensive evaluation of this association. Despite this constraint, it is acknowledged that women with PCOS exhibit elevated rates of obesity, insulin resistance (IR), and central fat deposition, factors that complicate the isolation of hyperandrogenism as an independent risk factor.^(2,4,17,18)^ For instance, in this study, only six women were classified as eutrophic, highlighting the challenge of isolating hyperandrogenism amidst the prevalent metabolic abnormalities characteristic of PCOS.

Another limitation of the present study pertains to the diagnostic methodology employed for NAFLD. Upper abdominal ultrasound was utilized, recognized as a primary screening tool for detecting steatosis. Nevertheless, liver elastography and magnetic resonance imaging are deemed superior diagnostic modalities, with liver biopsy serving as the gold standard.^(33)^

The notable prevalence of PCOS and the consequential impact of NAFLD on women's health serve as impetus for scientific inquiry into various clinical aspects associated with both pathologies. Consequently, screening for and early diagnosis of NAFLD in PCOS patients are imperative for mitigating disease progression and its resultant consequences.

## Conclusion

The prevalence of NAFLD among PCOS patients was 50.9%. Within this cohort, obesity and central fat deposition emerged as the primary factors associated with NAFLD, independent of age, race, and clinical and/or laboratory hyperandrogenism. Moreover, in the subset of patients afflicted with NAFLD, Metabolic Syndrome and alterations in liver enzymes exhibited a higher prevalence compared to those without the disease.
